# Does apocynin increase liver regeneration in the partial hepatectomy model?

**DOI:** 10.55730/1300-0144.5627

**Published:** 2023-05-31

**Authors:** Yılmaz BİLGİÇ, Burhan Hakan KANAT, Onural ÖZHAN, Azibe YILDIZ, Zeynep AKSUNGUR, Mehmet Erman ERDEMLİ, Nigar VARDI, Yusuf TÜRKÖZ, Sami AKBULUT, Adem KÖSE, Hakan PARLAKPINAR

**Affiliations:** 1 Division of Gastroenterology, Department of Internal Medicine, Faculty of Medicine, İnönü University, Malatya, Turkey; 2 Department of General Surgery, Faculty of Medicine, İnönü University, Malatya, Turkey; 3 Department of Pharmacology, Faculty of Medicine, İnönü University, Malatya, Turkey; 4 Department of Histology and Embryology, Faculty of Medicine, İnönü University, Malatya, Turkey; 5 Department of Biostatistics and Bioinformatics, Faculty of Medicine, İnönü University, Malatya, Turkey; 6 Division of Infectious Diseases, Department of Internal Medicine, Faculty of Medicine, İnönü University, Malatya, Turkey

**Keywords:** Antioxidant, apocynin, hepatectomy, liver regeneration, oxidative stress, rats

## Abstract

**Background/aim:**

Hepayocyte loss may develop secondary to liver surgery and at this point liver regeneration plays a significant act in terms of liver reserve. The purpose of this research was to investigate the efficacy of apocynin on liver regeneration and preservation after partial hepatectomy in rats.

**Materials and methods:**

A total of 32 rats, have been divided into 4 groups (n: 8) for hepatectomy model. Inflammatory and antiinflammatory parameters were measured from blood and liver tissue samples. In addition, the effects of apocynin were examined immunohistochemically and histopathologically from liver tissue.

**Results:**

In liver tissue samples, a significant difference has been found in glutathione peroxidase, total nitrite, catalase, oxidative stress index, total antioxidant and total oxidant status between sham and hepatectomy groups. A significant difference has been achieved between hepatectomy and posthepatectomy-Apocynin in terms of glutathione peroxidase and oxidative stress index. Total antioxidant status, oxidative stress index, and total oxidant status were significantly different only between the sham and the hepatectomy groups. Statistical differences were found between sham and hepatectomy groups and between hepatectomy and pre+post-hepatectomy-Apocynin groups in terms of serum glutathione, malondialdehyde, total nitrite, and L-Arginine. There were significant differences between the sham and hepatectomy groups, between hepatectomy and posthepatectomy-apocynin groups, between posthepatctomy-apocynin and pre+posthepatectomy-apocynin groups in terms of sinusoidal dilatation, intracytoplasmic vacuolization and glycogen loss (p < 0.001), in all histopathologic parameters except sinusoidal dilatation (p < 0.05). However, significant Ki–67 increases have been elaborated in hepatectomy, posthepatectomy-apocynin, and pre+posthepatectomy-apocynin groups compared to sham group (p < 0.001), in pre+posthepatectomy apocynin group compared to hepatectomy and posthepatectomy-apocynin groups (p < 0.001).

**Conclusion:**

Histopathology, immunohistochemistry, and biochemistry results of this study revealed that apocynin has a protective effect on enhancing liver regeneration in partial hepatectomy cases in rats.

## 1. Introduction

Partial hepatectomy of the liver (HP) is performed in benign and malignant tumors of the liver, abscess, cyst, trauma, and liver transplantation. In a healthy liver, mitosis is normally very low but regeneration increases after partial HP [1]. The rate actual rate of hepatocytes undergoing mitosis in healthy liver is 0.0012%–0.01% but it increases up to 3% after partial HP [2]. Liver regeneration can be elaborated as combined hyperplasia and hypertrophy of hepatocytes, matrix structures, and endothelium. Regeneration is controlled by genetic structure, various growth factors, and cytokines [3]. Inadequate liver regeneration increases morbidity and mortality, especially in liver transplantation, donor HP, and tumor resection [4]. If liver regeneration is insufficient, this can cause liver failure hence, insufficient triggering of cytokines and growth factors that stimulate liver regeneration may cause dysfunction in the residual liver. Another important problem is the incomplete regeneration as a result of these insufficient stimuli and the resulting fibrosis. By stimulating the regeneration in the liver with various methods, the development of undesirable events can be prevented. In this context, molecules that stimulate liver regeneration gain importance [5].

Apocynin is a molecule that has antiinflammatory, antioxidant, and apoptotic effects through various cytokines [6]. Studies with apocynin have shown neuro-protective, nephro-protective, pneumo-protective, and preventive effects against hypertension and therapeutic effects on colitis [6–9]. On the other hand, apocynin reduces ischemic reperfusion injury in the liver, as well as toxic damage caused by various factors via its protective effects [10–14]. Up to now, there have not any studies on the effects of apocynin on regeneration of liver. This study was conducted to investigate whether apocynin has an effect on liver regeneration in a partial HP animal model.

## 2. Materials & method

### 2.1. Ethical declaration

Ethics committee permission was obtained for the study (İnönü University Experimental Animal Research Ethics Committee, Malatya, Turkey) (protocol no: 2016/A–3, date: 15.04.2016).

#### 2.1.1. Chemicals

After dissolving apocynin (Sigma St, Louis, MO, USA) with 10% dimethyl-sulphoxide in serum physiologic solution as in the literature, it was administered intraperitoneally (IP) at a dose of 20 mg/kg [6–9].

#### 2.1.2. Animals

This study was performed on 32 male Sprague Dawley breeds aged between 11–12 weeks and weighing between 230–300 g, obtained from İnönü University Experimental Animal Research Center. The rats were fed with standard rat chow and housed in laboratory conditions (21 ± 2°C, 60% ± 5% humidity and 12:12 h light-dark cycle) where they could easily access water.

### 2.2. Study design

A total of 32 rats were used, four groups in total, with equal numbers of rats (n = 8) in each group. Groups were organized using the simple randomization method. Groups were defined as follows: Sham group (group 1), a group that was simply opened and reclosed without any treatment (rats received standard rat chow and water only). 0.5 mL vehicle solution (10% dimethyl-sulphoxide in serum physiologic saline) was administered IP once a day to all rats of sham group for 7 days. HP group (group 2), group with only partial HP (rats received standard rat chow and water only). 0.5 mL vehicle solution was administered IP once a day at to all rats of the partial HP group for 7 consecutive days after HP, starting 3 days before HP. Post-HP apocynin group (group 3), group administered 20 mg/kg apocynin as a single daily ip dose for 7 consecutive days after HP. Pre+post-HP apocynin group (group 4), group administered 20 mg/kg apocynin as a single daily ip dose for 7 consecutive days after HP, starting 3 days before HP. Partial HP was performed in all rats except sham group at 0. day. Simply opened and reclosed without partial HP operation was performed to sham groups at 0. day. All surgical procedures were performed under anesthesia, in which 100 mg/kg ketamine (Ketasol 10%; Richter Pharma Ag, Wels, Austria) and 10 mg/kg xylazine (XylazineBio 2%, Bioveta PLC, Ivanovice na Hane, Czech Republic) were administered IP. For pre+post-HP apocynin group, partial HP was performed at 0. day, but before HP, apocynin was applied to rats for 3 days. The days of the drug administration Pre- and Post-HP in the experimental protocol were designed according to the study of Kose et al. [15]. Partial HP was performed by sampling a piece of tissue from liver. All rats were weighed on the initiation of the experiment and just before sacrificing, and their weights were recorded. At the same time, liver weights on the day of HP and liver weights after sacrificing all rats except the first group were recorded. The weight change in rats during the study period was calculated. All rats were sacrificed with an administration of overdose (3 × anesthetic dose) injectable anesthetic agents 300 mg/kg ketamine and 30 mg/kg xylazine IP at the end of the eighth day of the end of the study. For biochemical analysis, blood was drawn from the inferior vena cava just before sacrification. HP was performed after blood collection and HP material was fixed with formalin 10% for histopathology analysis, the remaining material was stored at −70 °C for biochemical analysis.

### 2.3. Regeneration rate

The ratio of rat liver to rat total body weight was calculated using the sham group [16]. Whole liver weight was accepted as 3.4% of the rat weight [17]. Results were expressed as (%). Relative liver weight = [Liver weight at autopsy-(whole liver weight-weight of resected liver)/whole liver weight] × 100. Postoperative changes in body weight were measured at specified intervals. At various time points, rats were sacrificed by overdose of ketamine and xylazine cocktail (300 mg/kg and 30 mg/kg, IP), and residual liver weight was measured and the ratio of remaining liver weight to the initial body weight was calculated. The liver regeneration rate described by Fishback was calculated according to the following equation [16].

Liver Regeneration Rate (%) = 100 ×{C-(A-B)}/A. Where (A) is the estimated total liver weight at the time of operation. (B) is the weight of the excised liver. (C) is the residual liver weight at the time of death.

### 2.4. Biochemical analysis

Blood samples from rats were stored at −70 °C until testing. Liver samples were homogenized (IKA ultra turrax T 25 basic) in cold phosphate buffer (pH: 7.4, protease inhibitor cocktail, Bishop, 20 mmol) and centrifuged at 16,000 rpm for 3 min at +4 °C. Malondialdehyde (MDA) analysis was performed in homogenates. The remaining homogenates were centrifuged at 10,000 × g for 20 min at +4 °C and the resulting supernatants were total oxidant status (TOS), glutathione (GSH), glutathione peroxidase (GSH-Px) were used in the analysis of total antioxidant status (TAS), catalase (CAT), total nitrite (TN), and oxidative stress index (OSI) levels. Blood samples obtained from rats were centrifuged at 2000 × g for 10 min at +4 °C. TAS, GSH, TOS, TN, L-Arginine, OSI, asymmetric dimethylarginine (ADMA), symmetrical dimethylarginine (SDMA), and MDA levels were measured from serum samples.

#### 2.4.1. MDA measurement

Tissue levels of MDA, the most important indicator of lipid peroxidation, were determined by the analysis method of Uchiyama and Mihara [18]. The basic principle of the analysis is that when MDA is heated with thiobarbituric acid in an acidic environment, it reacts to form a pink-colored chromogen and the light intensity of this color was read in a spectrophotometer (Microplate reader; BioTek Synergy H1) at a wavelength of 532 nm and the amount of MDA is determined. The intensity of the pink color is directly proportional to the MDA concentration in the sample. Tissue MDA results are expressed as nmol/g wet tissue, and serum MDA results are expressed as μmol/L.

#### 2.4.2. GSH measurement

GSH levels of tissue samples were measured according to the method described by Ellman [19]. The GSH measurement principle is that the GSH reacts with 5,5′-dithiobis-2-nitrobenzoic acid. It gives a yellow-greenish color. The light intensity of this color is read in the spectrophotometer (Microplate reader; BioTek Synergy H1) at a wavelength of 410 nm and GSH amount is determined. The intensity of the yellow-greenish color is directly proportional to the GSH concentration in the sample. GSH results are expressed as nmol/g wet tissue in liver tissue and μmol/L in serum.

#### 2.4.3. CAT activity measurement

Measurement of CAT activity levels of tissue samples were performed according to Aebi’s analysis method [20]. Hydrogen peroxide (H_2_O_2_) is an absorbent substance in the ultraviolet spectrum and maximal absorbance occurs at 240 nm. The decomposition of H_2_O_2_ added to the test medium into water and oxygen by CAT is manifested by a decrease in absorbance at 240 nm. The CAT activity level is determined by reading this change in absorbance in a spectrophotometer (Microplate reader; BioTek Synergy H1) at a wavelength of 240 nm. This decrease in absorbance is directly proportional to the CAT enzyme activity in the medium. Tissue CAT results expressed K/g protein.

#### 2.4.4. GSH–Px activity measurement

Measurement of GSH–Px activity levels of tissue samples was performed according to the analysis method of Pagli and Valentine [21]. GSH-Px catalyzes the conversion of hydrogen peroxide from reduced GSH to water. At the end of the reaction, reduced GSH is oxidized. Another H_2_O_2_ catalyzes into water, by converting oxidized GSH into a reduced form. For the catalysis of another H_2_O_2_ to water, the oxidized GSH must be converted to the reduced form. This conversion is carried out in the presence of nicotinamide adenine dinucleotide phosphate hydrate (NADPH) and GSH reductase in the medium. In this case, NADPH is converted to NADP, while oxidized GSH is converted to reduced form. NADPH is a material that shows maximum absorbance at 340 nm. As GSH reductase catalysis continues, absorbance decreases at 340 nm as NADPH is converted into oxidized form. The GSH–Px activity level is determined by reading this change in absorbance at a wavelength of 340 nm in a spectrophotometer (Microplate reader; BioTek Synergy H1). This decrease in absorbance is directly proportional to the GSH–Px activity in the medium. Tissue GSH–Px results are expressed as U/g protein.

#### 2.4.5. TN measurement

Nitric oxide (NO) levels of serum and tissue supernatants were measured as TN by method of Jungersten et al. [22]. TN levels are accepted as the index of endogenous NO production [22, 23]. TN measurement was conducted according to the publication of Ozbek et al. [24]. Serum and supernatant samples from animals were deproteinized with ZnSO_4_ and NaOH. Then, 250 μL of deproteinized serum and supernatants were taken, 25 μL of nitrate reductase (10 U/mL, Sigma), 200 μL of 0.32 mol/L potassium phosphate buffer (pH 7.5). Flavine adenine dinucletide (FAD) (5 μmol/L) and NADPH (50 μmol/L) were added to a total of 750 μL of liquid containing 525 μL of distilled water and incubated for 2 h. After reduction of nitrate to nitrite by nitrate reductase, these reduced samples and Greiss reagent (greiss reagent) 0.1% α-naphthylamine dissolved in distilled water and 1% p-aminobenzene sulfamide dissolved in 5% phosphoric acid. It was prepared by taking a 1:1 ratio. After that the samples were left to incubate for another 15 min, absorbance values were read at 548 nm wavelength with the help of a spectrophotometer (Microplate reader; BioTek Synergy H1, USA).

Absorbance values were measured by preparing nitrite standards in a range of 0 to 100 μmol/L, and a standard graph was prepared. The absorbance values of the serum samples were converted to μmol/L nitrite using this standard plot. TN levels of tissue supernatants were expressed as nmol/g wet tissue and serum levels as μmol/L.

#### 2.4.6. Measurement of serum ADMA, SDMA, and L-Arginine levels

SDMA, ADMA, and L-Arginine in serum were measured by high performance liquid chromatography with commercial kits of the Eureka brand (Eureka Laboratory Department, Chiaravalle. Italy). Measurement results are expressed in μmol/L.

#### 2.4.7. Measurement of serum TAS and supernatant levels

Total antioxidant capacities of serum and tissue supernatants were measured spectrophotometrically at 660 nm in a microplate reader (BioTek Synergy H1) using the Rel Assay (Diagnostic, Turkey) commercial kit. In this method, the blue-green colored reduced 2,2′-azinobis, 3-ethylbenzothiazoline-6-sulfonate (ABTS) molecule is oxidized to the colorless ABTS+ cation in the presence of H_2_O_2_. Depending on the antioxidant concentration in the sample, this color loss is accelerated. This reaction is calibrated with the vitamin E analog Trolox (6-hydroxy-2.5.7.8-tetramethylchroman-2-carboxylic acid) Equivalent Standard Antioxidant Solution [25]. Supernatant and serum TAS levels are expressed as mmol Trolox Equivalent/L.

#### 2.4.8. Measurement of supernatant and serum TOS levels

Total oxidant levels in serum and tissue supernatants were measured spectrophotometrically (Microplate reader; BioTek Synergy H1) at 530 nm using the commercial kit Rel Assay (Diagnostic, Turkey). The oxidants in the samples oxidize the ferro ion (Fe^+2^)-o-dianisidine complex to the ferric ion (Fe^+3^). Ferric ion forms an orange-colored compound with xylenol in acidic environment. The color intensity is directly proportional to the amount of oxidant molecules present in the sample [25]. Supernatant and serum TOS levels are expressed as μmol H_2_O_2_ Equivalent/L.

#### 2.4.9. OSI calculation

OSI is calculated by dividing TOS by TAS. The OSI calculation formula is as follows: OSI = TOS (μmol H2O2 eqv/L)/TAS (mmol Trolox eqv/L) [25].

### 2.5. Histopathological evaluation

The liver tissue samples were fixed in 10% formalin and then embedded in paraffin. Tissue samples were cut at 4 μm thickness and placed on slides. Hematoxylin-eosin (H-E) stain was used to evaluate the general structure of the liver, and periodic acid schiff (PAS) stain was used to evaluate the glycogen accumulation in the liver. Evaluation of liver damage in tissue samples was done semiquantitatively. In this evaluation, dilatation in the sinuses, vacuolization in the cytoplasm, and loss of glycogen in hepatocytes were used. Liver damage was graded 0–3. Grade 0 if there was no liver damage; liver damage was classified as grade 1 if the damage was ≤25%, grade 2 if liver damage was between 25%–50%, and grade 3 if liver damage was ≥50%.

#### 2.5.1. Immunohistochemical evaluation

For immunohistochemical evaluation, tissue samples were first deparaffinized, then rehydrated and boiled in antigen recovery solution (citrate buffer, pH 6.0) for 20 min in a pressure cooker and finally cooled at room temperature for 20 min. Tissue samples were washed in phosphate-buffered saline (PBS). Tissue sections were taken from endogenous peroxide blocks and washed with PBS using 3% H_2_O_2_ solution for 15 min at room temperature. Afterwards, protein blocks were applied to the tissue sections. The obtained tissue sections were incubated with ki-67 primary antibody (Thermo Scientific, rabbit polyclonal) for 60 min, then washed with PBS and biotinylated goat antipolyvalent applied for 20 min at room temperature and incubated for 20 min using streptavidin peroxidase. Staining was completed for 10 min using chromogen+substrate and the slides were counterstained with Mayer’s hematoxylin for 1 min. Finally, they were dehydrated by washing in tap water.

Immunostaining with ki-67 antibody was used to evaluate hepatocyte regeneration. Brown staining of nuclei of cells was considered Ki-67 positive. The number of nuclei staining with Ki-67 was counted by examining 30 microscopic fields for each preparation under a 40-objective lens.

The Leica DFC280 was used as a light microscope for the evaluation of tissue sections and a Leica Q Win Image Analysis system (Leica Micros Imaging Solutions Ltd., Cambridge, UK).

### 2.6. Statistical analysis

SPSS 17 statistical program was used to compare histological results. The Mann-Whitney U (Bonferroni) test was used to compare groups. It is expressed as the median (min-max) in the results of the data. P values of <0.05 were considered significant.

## 3. Results

### 3.1. Assessment of weight changes

At the end of the study, there was no significant difference among the groups in terms of weight change from baseline (p=0.784). Data on weight change were given in Table 1.

### 3.2. Biochemical measurements of liver tissue

Liver tissue MDA (p < 0.001), GSH (p < 0.001), CAT (p < 0.001), GSH–Px (p = 0.014), TN (p < 0.001), TAS (p < 0.001), TOS (p < 0.001), and OSI (p < 0.001) have been statistically significantly different between the groups. When the tissue CAT, TN, and TAS levels were examined, it was seen that there was a statistically significant difference only between the sham group and the HP group. In terms of tissue GSH–Px and OSI levels, statistically significant differences were found between sham and HP groups and between HP and post-HP apocynin groups. There was a statistical difference between the HP group and the other three groups on MDA level, but there was no difference among the other three groups. Regarding TOS level, it was determined that there was a statistical difference between HP and sham group and pre+post-HP apocynin and HP groups. However, there was no statistical difference between HP and post-HP apocynin groups. All results are elaborated in the Table 2.

### 3.3. Biochemical measurements of blood

Serum MDA (p < 0.001), GSH (p = 0.001), TN (p = 0.001), TAS (p < 0.001), TOS (p = 0.001), OSI (p < 0.001), L-Arginine (p = 0.001), ADMA (p = 0.001), and SDMA (p = 0.001) levels were statistically significant between the groups. TAS, TOS, and OSI levels were statistically different only between the sham group and the HP group. In terms of serum MDA, GSH, TN, and L-Arginine levels, statistical differences were found between sham and HP groups and between HP and pre+post-HP apocynin groups. ADMA and SDMA levels, were significantly different between the HP group and the other three groups, but there was no difference between the other three groups (Table 3).

### 3.4. Histopathologic evaluation

**Figure 1:**
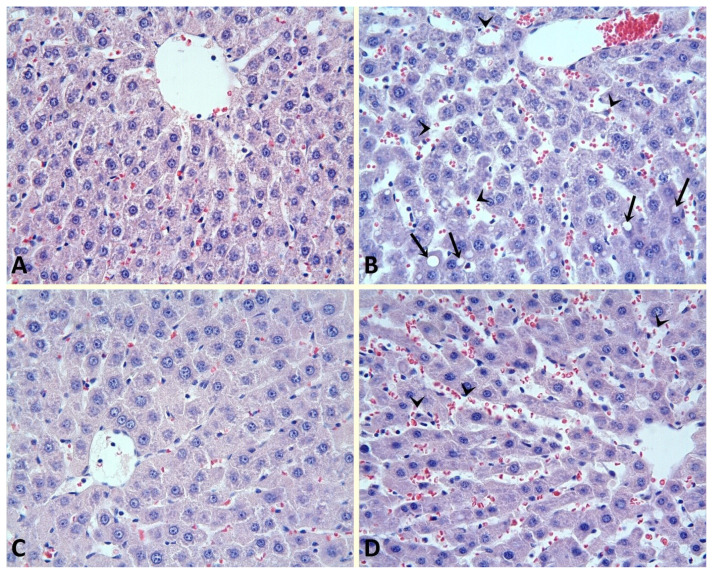
Sinusoidal dilatation and intracytoplasmic vacuolization in hepatocytes. Sham group (A); Normal histologic appearance of the liver. HP group (B); Sinusoidal dilatation (arrow heads) and intracytoplasmic vacuolization (arrows) are evident. Post-HP Apocynin group (C); Note that there is a similar appearance to the sham group. Pre+post-HP Apocynin group (D); Sinusoidal dilatation is still present (arrow heads). HEx40. HP: Hepatectomy.

Tissue samples from SH rats showed normal liver histology by the H-E dye method (Figure 1A). Sinusoidal dilatation and intracytoplasmic vacuolization were observed in the HP group (Figure 1B). There was a significant improvement in histological damage in the post-HP apocynin group (p < 0.05) (Figure 1C). The improvement in liver damage was more pronounced in the post-HP apocynin group than in the pre+post-HP apocynin group (p < 0.05) (Figure 1D).

**Figure 2:**
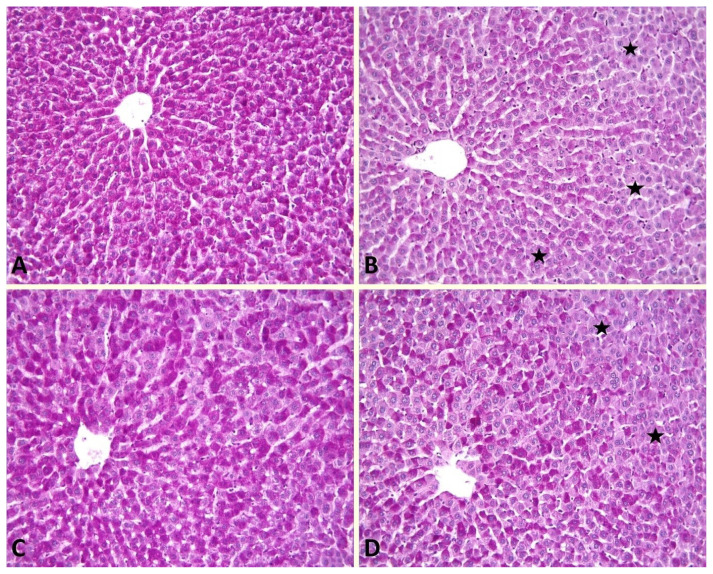
Glycogen storage in hepatocytes. Sham group (A); Hepatocytes containing glycogen exhibit magenta staining. HP group (B); Marked decreasing glycogen storage in hepatocytes (asteriks). Post-HP Apocynin group (C); Deposition of glycogen in hepatocytes is evident. Pre+post-HP Apocynin group (D); Notice a slight decrease in glycogen loss in hepatocytes (asteriks). PASx20. HP: Hepatectomy.

In the sham group, glycogen-containing cells showed magenta staining when stained with the PAS staining method (Figure 2A). In the HP group, it was observed that glycogen storage in hepatocytes decreased (p < 0.05) (Figure 2B). It was observed that apocynin treatment decreased glycogen loss in hepatocytes compared to the HP group (p < 0.05) (Figure 2C). It was observed that the decrease in glycogen turnover was more pronounced in the post-HP apocynin group compared to the pre+post-HP apocynin group (p < 0.05) (Figure 2D) (Table 4).

#### 3.4.1 Immunohistochemistry

**Figure 3:**
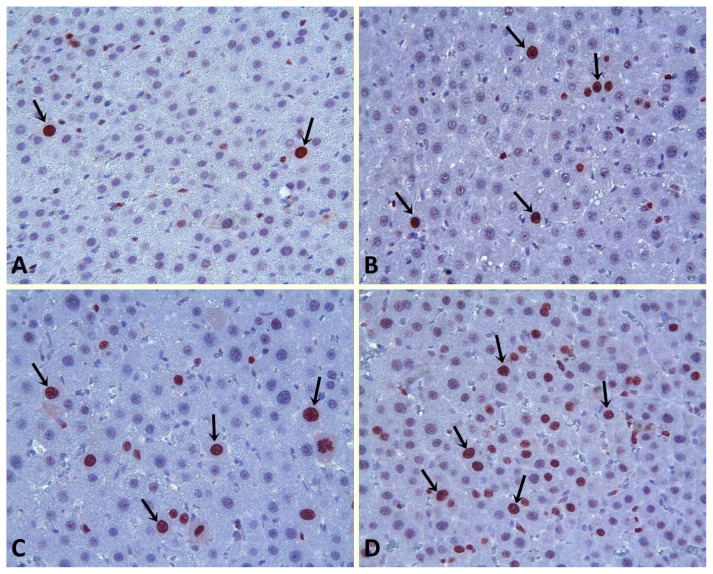
Arrows show the Ki–67 positive hepatocytes. Sham group (A); HP group (B); Post-HP Apocynin group (C); Pre+post-HP Apocynin group (D). Ki-67 immune staining ×40. HP: Hepatectomy.

Hepatocyte regeneration was evaluated with ki-67 from immune histochemical staining. It was observed that the excretion of ki-67 was significantly increased in the HP group compared to the sham group (p < 0.05). Ki-67 staining was found to be similar in the post-HP apocynin group compared to the HP group (p > 0.05). The number of Ki-67 positive cells was found to be higher in the pre+post-HP apocynin group than in all other groups (p < 0.05) (Figure 3A, 3B, 3C, and D).

### 3.5. Regeneration rate

The mean ratio of whole liver weight to body weight was 4.5% ± 0.07% (mean + SD; n = 6) therefore, using this value, an estimated full liver weight was obtained at the time of the operation. Whole liver weight was found to be 5.67% of the rat weight. When the calculated Fishback regeneration rates were compared between the groups, although there was an increase in apocynin areas, no statistically significant difference was observed (p < 0.05) (Table 5).

## 4. Discussion

In the present study, apocynin, which has antiinflammatory, antioxidant, and hepatoprotective effects, increases mitotic activity and liver regeneration in hepatocytes, while reducing oxidative stress after HP. According to our knowledge, this circumstance has been denoted for the first time in the literature.

Studies with apocynin have shown that in ischemia-reperfusion injury, apocynin induces the antioxidant activity by increasing GSH and reducing the damage [13]. An important enzyme in the formation of reactive oxygen substrates (ROS) because of oxidative stress is NADPH oxidase. In a study, it was shown that apocynin suppressed ROS formation by inhibiting NADPH oxidase [26]. In another study, it was reported that apocynin reduced free oxygen radicals with an antioxidant mechanism and showed a liver protective effect both in the serum of the patient and histopathologically in liver damage caused by cisplatin [14]. In another study, it was shown that APO treatment was protective against radiation-induced hepatic injury by decreasing oxidative stress and increasing antioxidant activity [27]. In a study, it was indicated that LPS/D-Gal exposure time-dependently increased the level of ROS in liver tissue [28]. A study showed that the combination of apocynin and α-LA has pronounced antifibrotic effects [29]. Previously, it was reported that ROS formed in the body continuously has detrimental effects on liver regeneration. It shows this negative effect by inhibiting cell cycle and growth stimuli involved in liver regeneration [30]. In the present study, it was confirmed that apocynin has an antioxidant effect both biochemically and histopathologically, in accordance with the literature. In this respect, it is thought that it contributes positively to liver regeneration by reducing ROS, which negatively affects liver regeneration as mentioned above.

Recently, it has been reported that oxidative stress occurs with the increase of ROS after liver surgery. Based on this relationship, it has been shown that presurgical immune nutrition improves postoperative complications by acting as an antioxidant [31]. One of the reasons for insufficient liver regeneration after HP and liver resection is the apoptotic cell death mechanism(s). It has been shown that the increase in ROS after such surgeries affects apoptosis. Apoptosis also impairs liver functions [32]. Apocynin pharmacokinetics and pharmacodynamics are characterized by the inhibition of ROS production in major organs [33]. In a study, it was demonstrated that ROS plays an important role in mediating Graft-Versus-Host Disease [34]. One of the important parameters showing liver regeneration is the number of Ki–67 in the cells. Increased Ki–67 is directly proportional to liver regeneration. In a study, it was shown that methylglyoxal induces ROS, significantly reduces Ki–67 expressions, and ultimately inhibits apoptosis and cell proliferation [32]. In our study, it was clearly demonstrated that apocynin did not increase liver regeneration in the calculation of the Fischbach method, but increased the expression of Ki–67, which was a histopathological method (increased regeneration), and stimulated it in the calculation by the HP method.

Due to the fact that it is an animal study, the short waiting period for the evaluation of regeneration with a limited number of subjects is among the limitations of the current study. Another limitation of the study was that the dose effect has not been evaluated by applying different doses of apocynin.

The fact that the results obtained in our study belong to an animal model prevents their clinical adaptation to humans. These results need to be confirmed by clinical studies in humans. There is a need for studies at the cellular level to evaluate the molecular mechanisms by which apocynin triggers regeneration in the liver.

## 5. Conclusion

To our knowledge, this was the first study in the literature on liver regeneration after partial HP. Therefore, the results of this study need to be supported by future research. In conclusion, in this experimental study model, it has been shown that apocynin has a beneficial effect on liver regeneration by showing antioxidant and antiapoptotic effects.

## Figures and Tables

**Table 1:** Changes in body weight in the groups.

Groups (mean ± SD)	Baseline weight (g)	Final weight (g)^a^	Change in weight (%)^b^
Sham	220.4 ± 13.0	217.0 ± 18.0	−1.41 ± 7.88
HP	273.5 ± 29.1	265.2 ± 33.5	−2.80 ± 8.80
Post-HP Apocynin	264.8 ± 30.5	260.6 ± 23.7	−1.47 ± 7.36
Pre+post-HP Apocynin	226.2 ± 18.6	215.3 ± 18.0	−4.39 ± 6.12

a Weights of resected liver from each rat were added to final weights of the rats.

b There was no significant difference between groups in regard of weight changes (p = 0.784). SD: Standard deviation, HP: Hepatectomy.

**Table 2:** Analysis of biochemical parameters in the liver tissues.

Parameters	Sham	HP	Post-HP Apocynin	Pre+post HP Apocynin	(p)
MDA (nmol/g wet tissue), median (min-max, IQR)	784 a (700–1039)(288)	1432 a,b,c (1390–1611) (134)	867 b (764–992) (165)	940 c (811–1039)(100)	<0.001
GSH (nmol/g wet tissue), median (min-max, IQR)	1602 d (1519–1897)(330)	936 a,d (820–1231)(160)	1650 a (1596–1891)(187)	1529 (1295–1840)(242)	<0.001
Catalase (K/g protein), median (min-max, IQR)	61^a^ (50–71)(12)	23^a^ (17–27)(7)	31(26–46)(9)	31 (26–56)(13)	<0.001
GSH-Px (U/g protein), median (min-max, IQR)	123^e^ (109–135)(21)	91^a^,^e^ (62–116)(16)	102a(74–156)(29)	102 (62–156)(44)	0.014
TN (nmol/g wet tissue), median (min-max, IQR)	288 f (263–313)(38)	166 f (148–176)(11)	299(274–342)(30)	274 (266–295)(29)	<0.001
TAS (mmol Trolox eqv/L), median (min-max, IQR)	2.00^a^ (2.00–2.10)(0.07)	1.26^a^ (1.00–1.40)(0.10)	1.85 (1.68–2.00)(0.13)	1.88 (1.74–1.98)(0.20)	<0.001
TOS (μmol H_2_0_2_ eqv/L), median (min-max, IQR)	21.4^b^ (19.5–24.0)(2.9)	32.2^b^,^g^ (30.8–35.2)1.7)	19.1 (12.7–27.0)(8.3)	22.9^g^ (18.0–26.9)(5.2)	<0.001
OSI (μmol H_2_0_2_ eqv/L)/mmol Trolox eqv/L) median (min-max, IQR)	10.4^a^ (9.7–11.5)(1.0)	26.0 a,h (22.0–31.0)(4.2)	10.6^h^ (6.9–15.0)(4.8)	12.9 (10.0–14.0)(3.3)	<0.001

a p < 0.001,

b p = 0.003,

c p = 0.042,

d p = 0.001,

e p = 0.01,

f p = 0.006,

g p = 0.031,

h p = 0.02.

MDA: Malondialdehyde, GSH: Reduced glutathione, GSHPx: Glutathione peroxidase, TN: Total nitrite, TAS: Total antioxidant status, TOS: Total oxidant status, OSI: Oxidative stress index, IQR: Interquartile range, HP: Hepatectomy.

**Table 3:** Analysis of biochemical parameters in the blood.

Parameters	Sham	HP	Post-HP Apocynin	Pre+post HP Apocynin	(p)
MDA (μmol/L), median (min-max, IQR)	6.20^a^ (5.60–7.20)(0.99)	8.40^a^,^b^ (7.90–9.10) (0.74)	7.10 (6.50–7.70)(0.73)	7.10^b^ (6.40–7.80)(1.05)	<0.001
GSH (μmol/L), median (min-max, IQR)	21.30 a (19.10–22.60)(2.98)	13.60 a,c (12.40–17.10)3.15)	18.50 (16.80–20.30)(1.66)	19.20^c^ (16.80–20.50)(2.89)	0.001
TAS (mmol Trolox eqv/L) median (min-max, IQR)	2.10^a^ (1.80–2.20)(0.30)	0.90^a^ (0.80–0.97)(0.14)	1.60 (1.50–1.70)(0.16)	1.80 (1.70–2.00)(0.16)	<0.001
TOS (μmol H_2_0_2_ eqv/L), median (min-max, IQR)	25.6 a (16.8–26.8)(4.9)	46.8 a (39.2–52.8)(10.4)	29.6 (24.8–32.0)(51.0)	30.2 (24.0–40.1)(9.6)	0.001
OSI (μmol H_2_0_2_ eqv/L)/mmol Trolox eqv/L) median (min-max, IQR)	12.30^a^ (8.00–14.70)(3.54)	49.20^a^ (40.00–60.00)8.14)	18.60 (14.80–20.00)(3.16)	17.20 (12.60–20.80)(5.25)	<0.001
TN (μmol/L), median (min-max, IQR)	19.0 d (17.0–20.0)(2.3)	13.7 d,e (13.0–15.0)(1.3)	17.0 (16.0–19.5)(2.0)	17.5 e (15.7–20.7)(2.0)	0.001
L-Arginine (μmol/L), median (min-max, IQR)	5.50 f (5.10–6.30)(0.75)	9.10 e,f (7.80–9.60)(1.50)	6.70 (5.20–9.30)(2.38)	6.00 e (4.40–8.10)(1.90)	0.003
ADMA (μmol/L), median (min-max, IQR)	0.30^h^ (0.30–0.40)(0.03)	0.60 d,g,h (0.50–0.70)(0.10)	0.25 d (0.20–0.30)(0.10)	0.30 g (0.20–0.40)(0.20)	0.001
SDMA (μmol/L), median (min-max, IQR)	0.50 l (0.50–0.60)(0.03)	0.80 g,k,l (0.80–0.90)(0.10)	0.50 k (0.40–0.60)(0.13)	0.50 g (0.40–0.60)(0.13)	0.001

a p < 0.001,

b p = 0.043,

c p = 0.05,

d p = 0.001,

e p =0.023,

f p = 0.04,

g p = 0.016,

h p = 0.049,

k p = 0.002,

l p = 0.015.

MDA: Malondialdehyde, GSH: Reduced glutathione, TN: Total nitrite, TAS: Total antioxidant status, TOS: Total oxidant status, OSI: Oxidative stress index, ADMA: Asymmetrical dimethyl arginine, SDMA: Symmetrical dimethyl arginine, IQR: Interquartile range, HP: Hepatectomy.

**Table 4:** Results of histopathological scores.

Groups [median, (min-max)]	Sinusoidal dilatation	Intracytoplasmic vacuolization	Glycogen loss
Sham	0.0 (0.0–1.0)	0.0 (0.0–0.0)	0.0 (0.0–2.0)
HP	1.0 (0.0–3.0)^a^	0.0 (0.0–3.0)^a^	2.0 (1.0–3.0)^a^
Post-HP Apocynin	0.0 (0.0–1.0)^b^,^c^	0.0 (0.0–0.0)^b^,^c^	1.0 (0.0–3.0)^b^,^c^
Pre+post-HP Apocynin	1.0 (0.0–3.0)^f^	0.0 (0.0–3.0)^d^	2.0 (0.0–3.0)^e^

a Significant increase in the HP group compared with the Sham group, (p < 0.0001);

b Significant decrease in post-HP Apocynin group compared with HP group, (p < 0.0001);

c Significant decrease in post-HP Apocynin group compared with pre+post-HP Apocynin group, (p < 0.0001);

d Significant decrease in pre+post-HP groups compared with HP group, (p < 0.0001);

e Significant decrease in pre+post-HP groups compared with HP group, (p = 0.003);

f Difference between HP and pre+post-HP Apocynin groups is not significant, (p = 0.549). HP: Hepatectomy.

**Table 5:** Ki–67 positive cell number and Fishback regeneration rate.

Groups [median, (min-max)]	Ki-67 (+) cells	Fishback regeneration rate^d^
Sham	1.0 (0.0–8.0)^a^	-
HP	3.0 (0.0–33.0)^b^	78.8 (56.9–94.1)
Post-HP Apocynin	5.0 (0.0–49.0)^c^	90.1 (82.8–105.5)
Pre+post-HP Apocynin	10.5 (0.0–56.0)	90.9 (74.1–178.7)

a Significant increase in HP, post-HP Apocynin and pre+post-HP groups compared with Sham group, (respectively, p < 0.0001, p < 0.0001 and p < 0.0001);

b Significant increase in pre+post-HP Apocynin group compared with HP group, (p < 0.0001); Difference between HP and Post-HP Apocynin groups is not significant, (p = 0.153);

c Significant decrease in post-HP Apocynin group compared with pre+post-HP Apocynin group, (p< 0.0001).

d There were no significant differences between groups in regard to Fishback regeneration rate (p < 0.05). HP: Hepatectomy.
